# CT of the chest with model-based, fully iterative reconstruction: comparison with adaptive statistical iterative reconstruction

**DOI:** 10.1186/1471-2342-13-27

**Published:** 2013-08-09

**Authors:** Yasutaka Ichikawa, Kakuya Kitagawa, Naoki Nagasawa, Shuichi Murashima, Hajime Sakuma

**Affiliations:** 1 Department of Radiology, Mie University Hospital, 2-174 Edobashi, Tsu, Mie 514-8507, Japan

**Keywords:** Model-based iterative reconstruction, Adaptive statistical iterative reconstruction, Filtered back projection, Low-dose computed tomography, Radiation dose reduction, Chest

## Abstract

**Background:**

The recently developed model-based iterative reconstruction (MBIR) enables significant reduction of image noise and artifacts, compared with adaptive statistical iterative reconstruction (ASIR) and filtered back projection (FBP). The purpose of this study was to evaluate lesion detectability of low-dose chest computed tomography (CT) with MBIR in comparison with ASIR and FBP.

**Methods:**

Chest CT was acquired with 64-slice CT (Discovery CT750HD) with standard-dose (5.7 ± 2.3 mSv) and low-dose (1.6 ± 0.8 mSv) conditions in 55 patients (aged 72 ± 7 years) who were suspected of lung disease on chest radiograms. Low-dose CT images were reconstructed with MBIR, ASIR 50% and FBP, and standard-dose CT images were reconstructed with FBP, using a reconstructed slice thickness of 0.625 mm. Two observers evaluated the image quality of abnormal lung and mediastinal structures on a 5-point scale (Score 5 = excellent and score 1 = non-diagnostic). The objective image noise was also measured as the standard deviation of CT intensity in the descending aorta.

**Results:**

The image quality score of enlarged mediastinal lymph nodes on low-dose MBIR CT (4.7 ± 0.5) was significantly improved in comparison with low-dose FBP and ASIR CT (3.0 ± 0.5, p = 0.004; 4.0 ± 0.5, p = 0.02, respectively), and was nearly identical to the score of standard-dose FBP image (4.8 ± 0.4, p = 0.66). Concerning decreased lung attenuation (bulla, emphysema, or cyst), the image quality score on low-dose MBIR CT (4.9 ± 0.2) was slightly better compared to low-dose FBP and ASIR CT (4.5 ± 0.6, p = 0.01; 4.6 ± 0.5, p = 0.01, respectively). There were no significant differences in image quality scores of visualization of consolidation or mass, ground-glass attenuation, or reticular opacity among low- and standard-dose CT series. Image noise with low-dose MBIR CT (11.6 ± 1.0 Hounsfield units (HU)) were significantly lower than with low-dose ASIR (21.1 ± 2.6 HU, p < 0.0005), low-dose FBP CT (30.9 ± 3.9 HU, p < 0.0005), and standard-dose FBP CT (16.6 ± 2.3 HU, p < 0.0005).

**Conclusion:**

MBIR shows greater potential than ASIR for providing diagnostically acceptable low-dose CT without compromising image quality. With radiation dose reduction of >70%, MBIR can provide equivalent lesion detectability of standard-dose FBP CT.

## Background

Radiation associated with diagnostic computed tomography (CT) has recently come under scrutiny because of the known association between ionizing radiation and malignancy. The lifetime cancer risk based on current CT use has been estimated to be as high as 2.0% [1]. There is a compelling need for high quality CT images acquired with reduced radiation doses.

The filtered back projection (FBP) technique is currently the most widespread CT reconstruction algorithm. However, this reconstruction technique does have significant limitations, mainly because it relies on several assumptions. The FBP technique assumes that there is a focal point source on the anode, a pencil-shaped beam emerging from the anode, a point-like interaction of the beam with the voxel, and a point-like interaction of the beam with the detector [2]. In actuality, all these assumptions about the x-ray beam are incorrect. These assumptions lead to substantial limitations in spatial resolution and noise generation. Furthermore, there is no general statistical consideration for noise. As a result, FBP images are prone to high levels of noise, streak artifacts, and low-contrast detectability in low-dose acquisitions [2].

Recently, adaptive statistical iterative reconstruction (ASIR) was introduced as a way to reduce image noise [3-6]. With this technique, projection data are first reconstructed with a FBP, and are then compared with an ideal noise model until the algorithm converges. Previous studies have demonstrated that there is a significant reduction in image noise, and, on average, a 25-50% dose reduction can be achieved [3,6-9]. One limitation, however, is that the ASIR technique continues to assume an ideal x-ray system.

Model-based iterative reconstruction (MBIR) is the most advanced of the various iterative reconstruction schemes as it attempts to model the entire x-ray beam as it travels from the cathode to the detector [10]. This includes modeling of the shape of the focal spot on the anode, the shape of the beam as it emerges from the anode, the 3-dimensional interaction of the beam with the voxel in the patient, and the 2-dimensional interaction of the beam with the detector [2]. By modeling these optical effects, MBIR can substantially improve image quality and spatial resolution, and reduce streaking artifacts. Recent studies demonstrated that low-dose MBIR images had significantly lower image noise than low-dose ASIR images [11,12]. However, the lesion detectability of low-dose MBIR CT has not been sufficiently studied yet. The purpose of this study was to evaluate the lesion detectability of low-dose chest CT reconstructed with MBIR and ASIR in comparison with standard-dose FBP CT.

## Methods

### Patients

This study was approved by the local institutional review board (Mie University Medical Research Ethics Committee). Informed consent was obtained from all participating patients. The study prospectively enrolled 55 patients (mean age, 72 ± 7 years; male / female = 25 / 30) who were referred for chest CT examinations between February 2011 and June 2011 because of suspected lung disease on chest radiograms. Patients were considered ineligible if they were younger than 60 years old. The mean body mass index (BMI) was 22.1 ± 3.4 kg/m^2^.

### CT scanning protocol

All 55 subjects underwent standard-dose and low-dose chest CT with a 64-section multi-detector row CT scanner (GE Discovery CT750 HD; GE Healthcare, Milwaukee, WI). A weight-based adjustment of a combined modulation type (Auto mA 3D) automatic exposure control technique was used for both CT scans. For standard-dose CT, a noise index of 18.33 was employed on the basis of vendor recommendation. For low-dose CT, a noise index of 36.66 was used to reduce the radiation dose by approximately 75% compared with standard-dose CT. With the exception of the noise index, all remaining scanning parameters were held identical for standard-dose and low-dose CT examinations. These parameters included helical scanning mode, 0.5 second gantry rotation time, minimum and maximum mA of 75 and 740, respectively, 120 kVp, 0.984:1 beam pitch, and a 40 mm table feed per gantry rotation. Standard-dose CT images were reconstructed with FBP using standard and bone kernels. Low-dose CT images were reconstructed with the FBP and ASIR technique using standard and bone kernels, and the MBIR algorithm. A blending factor of 50% was used for ASIR. All images are reconstructed with a 0.625 mm slice thickness which is standard for reading high resolution CT of the chest in our institute. Each image data set was coded, patient information was removed, and the sets were randomized by a study coauthor (N.N., with 12 years of experience) to enable double-blinded evaluation. All images were transferred to a commercially available workstation (Advantage Windows 4.2; GE Healthcare). A 21.2” color monitor with 1536 × 2048 of resolution was used for evaluation.

### Assessment of lesion detectability

First, one thoracic radiologist (S.M. with 25 years of experience) evaluated the presence or absence of abnormal structures in the lung and mediastinum on standard-dose FBP CT. Abnormal lung structures were assessed in four categories: consolidation or mass, ground-glass attenuation, reticular opacity, and decreased lung attenuation (bulla, emphysema, or cyst). The abnormal mediastinal structures were assessed in one category: lymph node enlargement (>1 cm along the minor axis).

Then, two thoracic radiologists (Y.I., with 14 years of experience, and K.K., with 15 years of experience) were asked to evaluate the image quality of those abnormal structures by consensus. Each image series was displayed in the blinded and randomized manner. The observers were previously informed of the presence and location of the lesions in the lung and mediastinum for evaluation. The radiologists were not aware of the clinical information, patient data, or image reconstruction techniques. Visualization of abnormal structures was evaluated on a 5-point scale (5 = excellent image quality with demarcation of structures, 4 = slight increase in noise or artifact, 3 = moderate increase in noise or artifact, 2 = severe increase in noise or artifact, and 1 = not applicable for the evaluation). Images were displayed in the lung image setting (window level, -500 HU; window width, 1500 HU) and in the mediastinal image setting (window level, 40 HU; window width, 350 HU) for evaluation. The observers were allowed to change the window width and window level and to use the pan/zoom functions.

### Image noise analysis

Objective assessment of image noise was performed by measuring the standard deviation of pixel values in homogeneous regions-of-interest (ROI) within the descending aorta at the level of the ventricular cavities on the standard kernel images. Care was taken to avoid superimposition of the ROI on the inner portion of the aortic wall.

The visual perception of noise, defined by a grainy appearance of the CT images, was evaluated by two thoracic radiologists (Y.I., with 14 years of experience, and K.K., with 15 years of experience) who were not aware of any clinical information or image reconstruction techniques. Images were displayed in the lung image setting (window level, -500 HU; window width, 1500 HU) and in the mediastinal image setting (window level, 40 HU; window width, 350 HU) for evaluation. Both readers evaluated image quality for the CT images reconstructed with the FBP, ASIR, and MBIR techniques by consensus. On each series of lung and mediastinal images, the image noise was graded on a 5-point scale (5 = minimum, 4 = less than average noise, 3 = average noise with an acceptable image, 2 = above average noise, and 1 = unacceptable image noise).

### Radiation dose analysis

To assess the radiation dose associated with the chest CT examinations, the total dose-length product, which represents the total absorbed dose for all the scan series, was recorded. Estimated effective doses were calculated from the total dose-length product using a revised normalized effective dose constant of 0.014 [13].

### Statistical analysis

Data were recorded on worksheets (Excel; Microsoft, Redmond, WA) and analyzed using Excel and SPSS for Windows, version 19 (SPSS, Inc, Chicago, IL). Continuous values are presented as mean ± standard deviation. Differences in objective image noise measurements, image noise scores, lesion conspicuity scores, and radiation dose were analyzed by the Wilcoxon signed-rank test. Differences by ages between male and female subjects were tested with the unpaired Student’s *t*-test. A *P* value of less than 0.05 was considered to represent a statistically significant difference.

## Results

There were no statistically significant differences associated with age in both male and the female patients in the present study (p = 0.05). Of the 55 patients evaluated, 45 patients (82%) had abnormal lung or mediastinal structures detected on chest CT. Abnormal structures were distributed as follows: areas of consolidation or mass in 27 patients, ground-glass attenuation in 22 patients, reticular opacity in 7 patients, areas of decreased lung attenuation (bulla, emphysema, or cyst) in 18 patients, and enlargement of mediastinal lymph nodes in 10 patients.

### Lesion conspicuity

**Figure 1:**
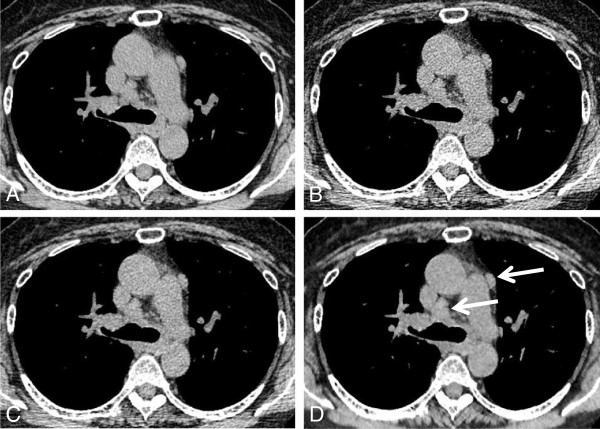
**Transverse chest CT through the ascending aorta in a 64 year-old woman with mediastinal lymph node enlargement (arrows).** Images were obtained with standard-dose FBP CT **(A)**, low-dose FBP CT **(B)**, low-dose ASIR 50% **(C)**, and low-dose MBIR method **(D)**. Note the excellent depiction of mediastinal lymph nodes on the low-dose MBIR image (lesion conspicuity score 5), compared with low-dose CT with FBP (score 3) and ASIR (score 4). Objective image noise on low-dose MBIR CT is 12.12 HU, showing higher than those on standard-dose FBP (17.72 HU), low-dose FBP (27.69 HU), and low-dose ASIR CT (20.76 HU) in this patient.

**Figure 2:**
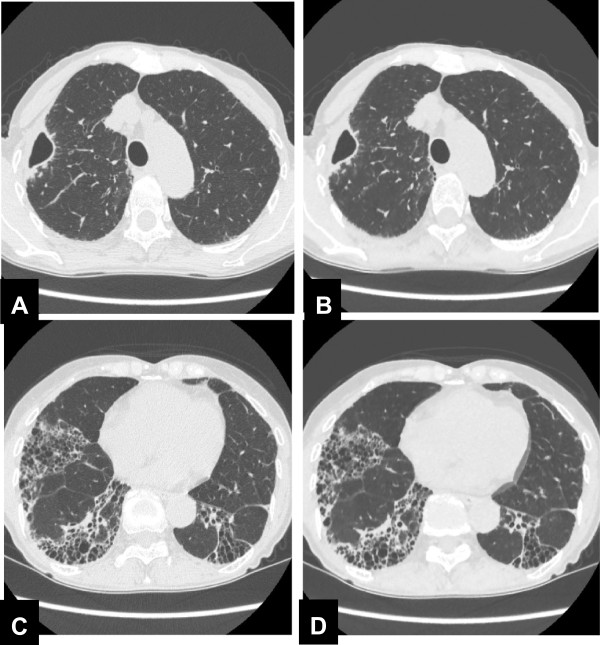
**Standard-dose FBP CT images at the level of aortic arch (A) and at the level of lower lung lobe (C), and low-dose MBIR CT images at the corresponding level (B and D) in a 67 year-old woman with lung cavities and reticular opacity.** Image quality score for those lesions on low-dose MBIR CT are both graded 5, showing equivalent on standard-dose FBP CT. Objective image noise measured is 13.04 on standard-dose FBP CT and 11.09 on low-dose MBIR CT in this patient.

Figure 1 shows a representative case with mediastinal lymph node enlargement. In Table 1, the results of lesion conspicuity on the chest CT are summarized. Concerning visualization of mediastinal lymph node enlargement, the image quality score on low-dose MBIR CT (4.7 ± 0.5) was significantly improved in comparison with low-dose FBP and ASIR CT (3.0 ± 0.5, p = 0.004; 4.0 ± 0.5, p = 0.02, respectively), and was nearly identical to the score of standard-dose FBP image (4.8 ± 0.4, p = 0.66). Image quality score of consolidation or mass, ground-glass attenuation, or reticular opacity on low-dose MBIR CT was 4.9 ± 0.2, 4.8 ± 0.4, and 5.0 ± 0, respectively, showing no significant differences in comparison with low-dose ASIR, low- and standard-dose FBP CT. As to areas of decreased lung attenuation (bulla, emphysema, or cyst), the image quality score on low-dose MBIR CT (4.9 ± 0.2) was slightly better compared to low-dose FBP and ASIR CT (4.5 ± 0.6, p = 0.01; 4.6 ± 0.5, p = 0.01, respectively). Figure 2 shows a comparison of image quality between standard-dose FBP and low-dose MBIR CT in a patient with lung cavities and reticular opacity. Low-dose CT with MBIR offers equivalent image quality compared with standard-dose FBP CT in this patient.

**Table 1:** Comparison of lesion conspicuity between filtered back projections and iterative reconstructions

	**Standard-dose CT with FBP**	**Low-dose CT with FBP**	**Low-dose CT with ASIR**	**Low-dose CT with MBIR**
Consolidation or mass	4.9 ± 0.2	4.7 ± 0.6	4.8 ± 0.4	4.9 ± 0.2
Ground-glass attenuation	5.0 ± 0.2	4.8 ± 0.4	4.8 ± 0.4	4.8 ± 0.4
Reticular opacity	5.0 ± 0	4.9 ± 0.4	5.0 ± 0	5.0 ± 0
Decreased lung attenuation (bulla, emphysema, or cyst)	4.9 ± 0.2	4.5 ± 0.6 *^§^	4.6 ± 0.5 *^§^	4.9 ± 0.2
Mediastinal lymph node enlargement	4.8 ± 0.4	3.0 ± 0.5 *^§^	4.0 ± 0.5 *^§^	4.7 ± 0.5

*CT* Computed tomography, *FBP* Filtered back projection, *ASIR* Adaptive statistical iterative reconstruction, *MBIR* Model-based iterative reconstruction.

The comparisons of lesion conspicuity scores were made by the Wilcoxon signed-rank test. * refers to statistically significant differences with standard-dose FBP CT (* p < 0.05). ^§^ refers to statistically significant differences with low-dose MBIR CT (^§^ p < 0.05).

### Image noise

**Figure 3:**
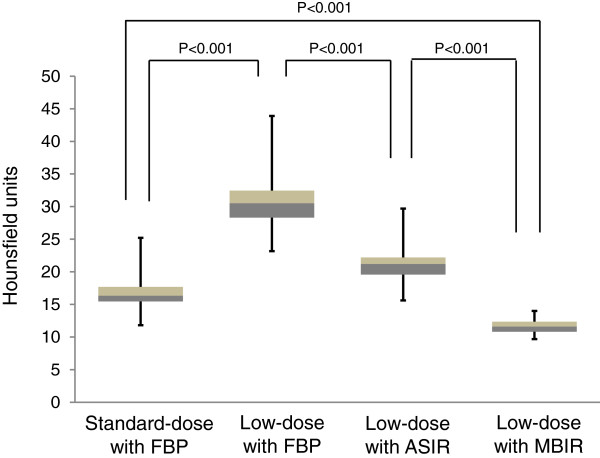
**Comparison of objective image noise measurements.** Images at the level of the descending aorta on the standard-dose CT with FBP and low-dose CT with FBP, ASIR, and MBIR. Low-dose MBIR CT demonstrated significantly reduced image noise in comparison with standard-dose FBP CT (p < 0.001) with a mean noise reduction of 29.3 ± 8.2%.

**Figure 4:**
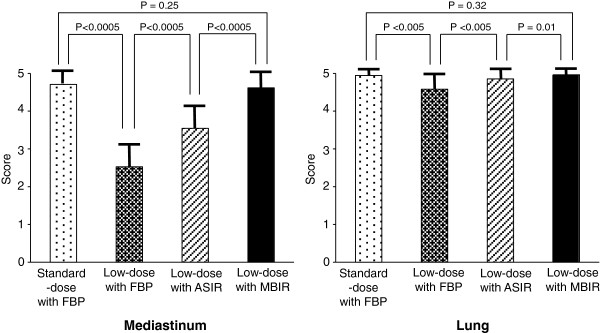
**Subjective image noise score.** Mediastinal and lung images of standard-dose CT aquired with FBP and low-dose CT with FBP, ASIR, and MBIR. The MBIR algorithm yields significantly improved image noise scores, especially of the mediastinal structures on low-dose chest CT.

In Figure 3, the mean values of objective noise measurements at the level of the descending aorta are summarized. Image noise on low-dose MBIR CT (11.6 ± 1.0 HU) were significantly lower than those on low-dose FBP CT (30.9 ± 3.9 HU, p < 0.001) and low-dose ASIR CT (21.1 ± 2.6 HU, p < 0.001), with mean noise reductions of 62.1 ± 3.3% and 44.5 ± 4.9%, respectively. In addition, low-dose MBIR CT demonstrated significantly reduced image noise in comparison with standard-dose FBP CT (16.6 ± 2.3 HU, p < 0.001) with a mean noise reduction of 29.3 ± 8.2%. The results of image noise scores on chest CT images are shown in Figure 4. There was a significant reduction in the level of subjective image noise on low-dose MBIR CT (4.6 ± 0.5) compared with that of low-dose FBP (2.5 ± 0.6, p < 0.001) and ASIR CT (3.5 ± 0.6, p = 0.01) on the mediastinal images. For the lung images, there was a slight but significant improve-ment in image noise with the MBIR technique (5.0 ± 0.2), compared with the FBP (4.6 ± 0.5, p < 0.001) and ASIR techniques (4.9 ± 0.4, p = 0.01) on low-dose CT. There were no significant differences in image noise scores on the lung images between low-dose MBIR and standard-dose FBP CT (5.0 ± 0.2 vs 4.9 ± 0.2, p = 0.32).

### Radiation dose

The mean dose-length product and estimated effective dose of low-dose chest CT was 112.5 ± 55.6 mGy∙cm and 1.6 ± 0.8 mSv, respectively. This was significantly lower than that of standard-dose chest CT (410.6 ± 165.2 mGy∙cm, p < 0.001; 5.7 ± 2.3 mSv, p < 0.0005). The mean percentage of radiation dose reduction was 73.3 ± 3.0% with the low-dose CT protocol.

## Discussion

The current prospective study demonstrated that MBIR techniques enabled an average of >70% radiation dose reduction compared with routine-dose FBP techniques for chest CT examinations without compromising diagnostic image quality. No significant differences were seen between low-dose MBIR and standard-dose FBP chest CT with regards to lesion detectability for lung consolidation or mass, ground-glass attenuation, or reticular opacity. On low-dose chest CT, the image quality of mediastinal lymph nodes and low attenuation lung diseases were significantly improved by MBIR techniques in comparison with ASIR techniques. Objective image noise on low-dose MBIR CT was significantly lower than that on low-dose ASIR CT.

Over the past several years, there has been a concerted effort to reduce radiation exposure in thoracic CT with various methods including tube current modulation, BMI-based tube voltage reduction, decreased scan length, low tube current scanning, and ASIR [3,6-9,14-22]. Recently developed MBIR is a more advanced and complex iterative reconstruction technique in compared with ASIR in that the reconstruction algorithm includes modeling of the x-ray optic system. Prior phantom studies have reported the potential of MBIR in reduction of image noise and artifacts [10,23,24]. A recent *ex vivo* study [25] demonstrated that the MBIR method leads to significantly decreased image noise accompanied with a substantial improvement of image quality and contrast-noise-ratio compared to the FBP and ASIR techniques. Katsura M, et al. [12] showed that low-dose MBIR chest CT images had significantly lower objective image noise (16.93 ± 3.00) than low-dose ASIR (49.24 ± 9.11, p < 0.01) and standard-dose ASIR images (24.93 ± 4.65, p < 0.01). In line with these previous studies, image noise with low-dose MBIR CT (11.6 ± 1.0 HU) in our study was significantly lower than that with low-dose FBP CT (30.9 ± 3.9 HU, p < 0.001) and low-dose ASIR CT (21.1 ± 2.6 HU, p < 0.001), with a mean noise reduction of 62.1 ± 3.3% and 44.5 ± 4.9%, respectively.

On the other hand, few data are available regarding its effect on diagnostic acceptability and lesion detectability on low-dose CT. We found that MBIR method is quite useful for improving image quality of mediastinal structures on low-dose chest CT. The present study demonstrated the lesion conspicuity score for mediastinal lymph node enlargement on low-dose MBIR CT (4.7 ± 0.5) was also significantly improved in comparison with low-dose FBP and ASIR CT (3.0 ± 0.5, p = 0.004; 4.0 ± 0.5, p = 0.02, respectively), and nearly identical to the conspicuity score for standard-dose FBP CT (4.8 ± 0.4, p = 0.66). The image quality score for decreased lung attenuation (bulla, emphysema, or cyst) on low-dose MBIR CT was slightly better than on low-dose FBP and ASIR CT, and similar to that on standard-dose FBP CT. Concerning visualization of consolidation or mass, ground-glass attenuation, or reticular opacity, there was no significant differences between low-dose MBIR CT and standard-dose FBP CT. Considering marked improvement of mediastinal image quality, low-dose MBIR CT can be alternative to standard-dose FBP CT. Although dose reduction is desirable for all patients, this new reconstruction algorithm may thus have significant impact in imaging young patients requiring CT, patients requiring serial CT follow-up, and the pregnant patients in whom imaging is deemed medically necessary. However, MBIR does have some drawbacks in the current setting. First, image processing is extremely slow. This is because MBIR is a complicated algorithm which uses multiple iterations and multiple models. Even with use of parallel processors, more than an hour is needed to process a typical 600-slice dataset. Because of this lengthy reconstruction time, initial application of this technique to clinical practice will mainly focus on patients with nonurgent or nonemergent conditions. Fortunately, in most practices, the majority of CT scans are performed in the outpatient setting, and immediate assessment is not mandatory. Furthermore, it is possible to have a preliminary set of ASIR images for immediate review. Second, the MBIR technique cannot be used for reconstruction of the electrocardiographic-gated CT images. It has already been reported that the ASIR technique can reduce image noise on coronary CT angiography [7]. Third, MBIR CT images have a slightly different “look and feel” compared with images reconstructed with FBP because images are reconstructed in a statistically optimal fashion. In this regard, Xu et al. recently raised a practical concern that statistical reconstruction might give an impression of somewhat reduced diagnostic value by radiologists who are used to FBP image appearance [26]. However, in the present study, standard dose FBP and low dose MBIR demonstrated equivalent diagnostic information. It is possible that interpreters can adapt themselves to the new look of MBIR in a relatively short period of time, particularly if they have preliminary experience with images from other iterative reconstruction techniques.

There were several limitations to the present study design. First, the sample size was small owing to the need for written informed consent for the additional radiation dose to participating patients. Second, image analysis was made by consensus between two readers and did not include assessment of inter- or intra-observer agreement between the two radiologists enrolled in this analysis. Because of the recent introduction of MBIR technique, this study design was considered a preliminary evaluation. Third, chest CT images with a noise index >36.33 were not assessed. It is possible that radiation doses of chest CT may be further decreased with MBIR. Fourth, for the ASIR method, a 50% blending factor was selected on the basis of vendor recommendations. It is conceivable that a higher ASIR percentage would allow even greater noise reduction and subsequent dose reduction. This idea needs to be balanced with concerns about loss of image detail with a higher degree of ASIR [6]. Fifth, the body size of the patients in this study was generally small. MBIR has not yet been assessed in extremely large or obese patients. Sixth, in this study, the observers evaluated the images in a blinded and randomized manner; however, they could recognize the reconstruction algorithms of the images to some extent because of differences in the appearances of the image data sets. This could be a potential source of observer bias.

## Conclusion

In conclusion, MBIR shows greater potential than ASIR for providing diagnostically acceptable low-dose CT without compromising image quality. With radiation dose reduction of >70%, MBIR can provide equivalent lesion detectability of standard-dose FBP CT.

## Abbreviations

ASIR: Adaptive statistical iterative reconstruction; CT: Computed tomography; FBP: Filtered back projection; MBIR: Model-based iterative reconstruction.

## Competing interests

The authors have no competing interests to declare.

## Authors’ contributions

Author contributions were as following; conception and design (YI, KK, NN, SM, and HS), analysis and interpretation of data (YI and KK), drafting of the manuscript (YI), and revising it critically for important intellectual content (KK, SM, HS). All authors have read and approved the final version of the manuscript.

## Pre-publication history

The pre-publication history for this paper can be accessed here:

http://www.biomedcentral.com/1471-2342/13/27/prepub
